# Supplementary material for: The Association of Moral Injury and Healthcare Clinicians’ Wellbeing: A Systematic Review
Source: Int J Environ Res Public Health. 2023 Jul 5;20(13):6300. doi: 10.3390/ijerph20136300 (PMC10341511; doi:10.3390/ijerph20136300)
Supplement: Supplementary file 1 [file ijerph-20-06300-s001.zip › ijerph-2304079-supplementary.pdf]

Supplement 1: *JBI Critical Appraisal Checklist for Qualitative Research Tool*

Authors (Year)	Is there congruity between the stated philosophical perspective and the research methodology?	Is there congruity between the research methodology and the research question or objectives?	Is there congruity between the research methodology and the methods used to collect data?	Is there congruity between the research methodology and the representation and analysis of data?	Is there congruity between the research methodology and the interpretation of results?	Is there a statement locating the researcher culturally or theoretically?	Is the influence of the researcher on the research, and vice-versa, addressed?	Are participants, and their voices, adequately represented?	Is the research ethical according to current criteria or, for recent studies, and is there evidence of ethical approval by an appropriate body?	Do the conclusions drawn in the research report flow from the analysis, or interpretation, of the data?
Alexander (2020) [44]	Yes	Yes	Yes	Yes	Yes	Yes; Partially	Yes	Yes	Yes	Yes
Ball et al. (2020) [45]	Yes	Yes	Yes	Yes	Yes	Yes; Partially	Unclear	Yes	Yes	Yes
Brown et al. (2021) [46]	Yes	Yes	Yes	Yes	Yes	Yes	Yes	Yes	Yes	Yes
Kreh et al. (2021) [18]	Yes	Yes	Yes	Yes	Yes	Yes; Partially	Unclear	Yes	Yes	Yes
Murray et al. (2018) [19]	Yes	Yes	Yes	Yes	Yes	No	No	Yes	Yes	Yes

Supplement 2: *JBI Critical Appraisal Checklist for Case Series (Longitudinal Studies)*

Author (Year)	Were there clear criteria for inclusion in the case series?	Was the condition measured in a standard, reliable way for all participants included in the case series?	Were valid methods used for identification of the condition for all participants included in the case series?	Did the case series have consecutive inclusion of participants?	Did the case series have complete inclusion of participants?	Was there clear reporting of the demographics of the participants in the study?	Was there clear reporting of clinical information of the participants?	Were the outcomes or follow up results of cases clearly reported?	Was there clear reporting of the presenting site(s)/clinic(s) demographic information?	Was statistical analysis appropriate?
Dale et al. (2021) [47]	Yes	Yes	Yes	Yes	Yes	Yes	Yes	Yes	Yes	Yes
Hines et al. (2021) [16]	Yes; Partially	Yes	Yes	Yes	Yes	Unclear	Not Applicable	Yes	Yes; Partially	Yes

Supplement 3: *JBI Critical Appraisal Tool for Cross-Sectional Studies Tool*

Authors (Year)	Were the criteria for inclusion in the sample clearly defined?	Were the study subjects and the setting described in detail?	Was the exposure measured in a valid and reliable way?	Were objective, standard criteria used for measurement of the condition?	Were confounding factors identified?	Were strategies to deal with confounding factors states?	Were the outcomes measured in a valid and reliable way?	Was the appropriate statistical analysis used?
Benatov, Zerach, & Levi-Belz (2022) [48]	No	Yes, Partially	Yes	Yes	Yes	No	Yes	Yes
Chandrabhatla et al. (2022) [49]	Yes	Yes, Partially	Yes	Yes	Yes	Yes	Yes	Yes
Levi-Belz & Zerach (2022) [50]	Yes	Yes, Partially	Yes	Yes	Yes	No	Yes	Yes
Litam & Balkin (2020) [51]	Yes	Yes	Yes	Yes	Yes	Unclear	Yes	Yes
Mantri et al. (2021)a [52]	Yes; Partially	Yes	Yes; Partially	Yes	Yes	No	Yes	Yes
Mantri et al. (2021)b [53]	Yes; Partially	Yes	Yes	Yes	Yes	Yes	Yes	Yes
Morris et al. (2022) [54]	Yes, partially	Yes	Yes	Yes	Yes	Yes	Yes	Yes
Ulusoy & Çelik (2021) [55]	Yes	Yes	Yes	Yes	Unclear	Unclear	Yes	Yes
Zerach & Levi-Belz (2021) [56]	Yes	Yes, partially	Yes	Yes	Yes	No	Yes	Yes
Zhizhong et al. (2021) [57]	Yes	Yes	Yes	Yes	Yes	Yes	Yes	Yes
Zhizhong, et al. (2020) [58]	Yes	Yes	Yes	Yes	Yes	No	Yes	Yes
